# Binge Eating Disorder and Metabolic Syndrome: Shared Mechanisms and Clinical Implications

**DOI:** 10.3390/healthcare13050482

**Published:** 2025-02-23

**Authors:** Michel Alagha, Firas Al-Alam, Karmen Saroufine, Linda Elias, Mark Klaimi, Ghassan Nabbout, Frederic Harb, Sami Azar, Nayla Nahas, Hilda E. Ghadieh

**Affiliations:** 1 Department of Biomedical Sciences, Faculty of Medicine and Medical Sciences, University of Balamand, Al-Koura, Tripoli P.O. Box 100, Lebanon; michel.alagha@std.balamand.edu.lb (M.A.); karmen.saroufine@std.balamand.edu.lb (K.S.); linda.elias@std.balamand.edu.lb (L.E.); ghassan.nabbout@fty.balamand.edu.lb (G.N.); frederic.harb@balamand.edu.lb (F.H.); sami.azar@balamand.edu.lb (S.A.); 2 Department of Psychology, Faculty of Arts and Sciences, University of Balamand, Al-Koura, Tripoli P.O. Box 100, Lebanon; firas.a.alam@fty.balamand.edu.lb (F.A.-A.); nayla.nahas@balamand.edu.lb (N.N.); 3 Department of Biology, Faculty of Arts and Sciences, University of Balamand, Al-Koura, Tripoli P.O. Box 100, Lebanon; mark.klaimi@std.balamand.edu.lb

**Keywords:** binge eating disorder, metabolic syndrome, obesity, genetic factors, behavioral factors, neurological factors, biological factors, food insecurity

## Abstract

**Background:** Binge eating disorder (BED) is characterized by episodes of uncontrollable eating, defined by the rapid consumption of large quantities of food over a short period. This condition is associated with a variety of psychological and non-psychological factors, including behavioral, biological, genetic, neurological, and pharmacological influences, all of which adversely affect patients’ daily lives. BED is linked to numerous health consequences, such as obesity, atherosclerosis, diabetes, chronic pain, and hypertension. Although BED is not exclusive to individuals with obesity, it is more prevalent in this population, who also face a heightened risk of developing metabolic syndrome (MetS). The latter is a cluster of five risk factors—obesity, hyperlipidemia, hyperinsulinemia, hypertension, and hyperglycemia—that significantly increase the likelihood of chronic diseases. **Methods**: This narrative review synthesizes existing research to explore the association between BED and MetS, examining shared pathophysiological mechanisms and clinical implications. It also highlights the role of escalating food insecurity and ongoing political, economic, and health crises in the development of BED. **Results**: BED is significantly associated with MetS components, including hypertension, obesity, type 2 diabetes, and dyslipidemia, all contributing to increased morbidity and mortality. Beyond body weight, behavioral, genetic, biological, and neurological factors mediate this relationship. **Conclusions**: BED is strongly linked to MetS through shared behavioral, genetic, and biological pathways. Early detection, integrated management strategies, and further research are crucial to addressing the public health challenges posed by this association.

## 1. Introduction

Binge eating disorder (BED) is characterized by the regularity of binge eating episodes [1]. They represent episodes of uncontrollable food consumption. During these episodes, individuals consume large amounts of food within a short specific time frame. For instance, food ingestion occurs within any two-hour period. Furthermore, individuals lose the ability to control their eating behaviors. Thus, binge eating episodes represent the core psychopathology that characterizes BED [2]; classified as an eating disorder. The Diagnostic and Statistical Manual of Mental Disorders, fourth edition (DSM-IV), published in 1994, included BED in the ‘Eating Disorders Not Otherwise Specified’ (EDNOS) category (American Psychiatric Association, 1994). This category acknowledged the existence of eating disorders that didn’t fully meet the criteria for established diagnoses at that time, but it was not a formal diagnosis by itself. Then, it was moved to the diagnosis section and classified in 2013 by the 5th edition of the Diagnostic and Statistical Manual of Mental Disorders (DSM-5) [3,4]. Then in 2018, BED was classified in the 11th edition of the International Classification of Diseases (ICD-11) by the World Health Organization (WHO). This classification acknowledged BED as a specific clinical diagnosis, distinct from other eating disorders. Table 1 highlights the diagnostic criteria for BED.

BED is a significant health concern, affecting a large portion of the population, and is frequently linked to obesity, with its occurrence documented at 30% in individuals pursuing surgical weight loss therapy [6]. BED transcends weight categories, affecting individuals of normal weight, overweight, and obese builds. Although it frequently appears alongside overweight and obesity in people seeking treatment, BED remains a distinct condition. Most obese individuals do not experience recurrent binge eating episodes. Research highlights this difference further. In controlled studies, people with BED consume more calories compared to weight-matched obese individuals without the disorder. Additionally, BED is linked to a greater struggle with daily functioning, a lower quality of life, and a higher burden of emotional distress and co-occurring mental health problems [1]. Research suggests a trend of increasing prevalence of BED along with increasing body mass index (BMI). While individuals with normal weight (under 25 BMI) may have BED, studies estimate the prevalence to be around 1.5% [7]. This prevalence appears to rise for overweight or obese individuals (25–30 BMI), with estimates suggesting rates around 5%. The highest prevalence estimates, ranging from 10–20%, are found among individuals with a BMI exceeding 30 [8]. It is important to remember that these are estimates, and actual prevalence rates can vary depending on several factors. Thus, while BED prevalence increases with BMI, a significant number of individuals with BED have a normal BMI [7].

The etiology of BED is complex and multifactorial, similar to other psychiatric disorders, with biological, individual, and societal factors all influencing dysregulated eating and related behaviors. BED may be characterized by abnormalities in reward processing, inhibitory control, and emotion regulation that fall along the spectrum of impulsivity [9]. The aim of this narrative review is to examine various factors contributing to the development of BED, and to provide a comprehensive overview of the current understanding of its complexity.

## 2. Metabolic Syndrome and Binge Eating Disorder

An increase in the risk of heart disease and cardiovascular disease (CVD) as well as diabetes mellitus has been associated with metabolic syndrome (MetS) linked with health problems such as hypertension, hypertriglyceridemia, low levels of high-density lipoprotein (HDL), hypertension, and fasting hyperglycemia [3,4]. Among adults in the US, the prevalence of the MetS includes the general population (25%) with obese individuals being at a higher risk [2]. The significance of coronary atherosclerosis, precisely in non-diabetic patients, is closely associated with several MetS components [3,4]. MetS has also been linked to lifestyle choices including smoking and physical inactivity as well as physiological traits like anger and despair. Additionally, insulin resistance has been documented as a fundamental mechanism for MetS. However, the pathogenic process of insulin resistance is not well understood [2].

Research has shown that MetS and BED share similar eating habits, such as consuming large amounts of food in a short period of time. This habit has been associated with lower glucose tolerance, as well as higher fasting glucose, serum fat, and insulin production levels. In such cases, individuals with obesity and BED exhibit higher serum cholesterol levels, fatty liver disease, and a larger waist-to-hip ratio. Recent longitudinal studies have indicated that this subgroup is more likely to develop MetS over time. Recognizing the correlations and prevalence of MetS in patients with obesity and BED is crucial [2].

According to laboratory research, BED patients’ typical eating habits may raise their chances of developing metabolic problems [5]. Furthermore, a cross-sectional investigation of the population found that irregular mealtimes were positively linked with the MetS [5]. Diagnosing patients with metabolic syndrome (MetS) required fulfilling specific criteria. A minimum of three out of five diagnostic criteria set by the National Cholesterol Program’s Adult Treatment Panel III (NCEP ATP III) guidelines. The criteria include high triglyceride levels, low HDL, and high blood pressure. Additional criteria include central obesity and an increase in fasting glucose levels. It has been estimated that obese patients with BED that meet these criteria constitute an estimate of 60%. Men and white individuals recorded higher rates compared to women and African Americans. Decreased episodes of weight cycling and regular meal skipping were potent predictors of MetS. This underscores the importance of weight loss promotion and regular meal patterns to prevent MetS and managing it in obese patients with BED. However, it is worth noting that binge eating frequency and eating disorder severity was of small difference between patients with MetS and those without. Thus, it is important to encourage weight loss and promote consistent meal patterns as strategies to prevent or manage MetS in obese individuals with BED [6,10]. Due to cultural and societal factors, it could be that men may not seek help until their condition has already caused significant physical or psychological distress.

## 3. Behavioral Factors Associated with Prevalence of MetS in BED Patients

Several behavioral factors have been associated with the occurrence of MetS in obese BED patients. For instance, an increase in the prevalence of MetS was recorded in obese BED patients undergoing regular meal skipping and weight cycling behavior [6]. These results were consistent with the outcome obtained by a previously conducted population-based sample [6]. Regular meal skipping refers to the habitual omission of planned meals throughout the day. This can involve intentionally skipping breakfast, lunch, or dinner, or a combination of these meals, and is distinct from occasional fasting or dietary practices that involve planned meal timing restrictions [6]. Weight cycling refers to the repeated pattern of weight loss and regain over time [11]. This cycle can result from intentional efforts, such as dieting or exercise, followed by weight regain, or from unintentional fluctuations in weight caused by various factors, including changes in lifestyle habits.

Regular meal skipping and weight cycling are considered potent predictors for BED and MetS, even after controlling for gender, BMI, and ethnicity. Such results were obtained through a multivariate hierarchical logistic regression study [6]. The relationship between regular meal skipping and MetS is important, as meal skipping is a modifiable behavior that patients are often encouraged to change. Regular meal consumption appears to be significantly associated with a reduced risk of MetS and other cardiovascular diseases [6]. Regular meal skipping and fewer episodes of weight cycling were important predictors of MetS. These results suggest that counseling patients to adopt regular eating patterns and focus on weight loss may have significant effects on the prevention and/or treatment of MetS in obese individuals with BED. [6].

## 4. HbA1c: Potential Association Between BED and MetS

There is a growing interest in the potential link between BED and HbA1c levels, a diagnostic indicator for diabetes [2,3,4,12]. An HbA1c of 6.5% is regarded as the cutoff for diabetes [5]. According to the American Diabetes Association, HbA1c levels between 5.7% and 6.4% are considered pre-diabetic. Doctors are reminded that while the risk of retinopathy increases significantly, by approximately 0.065, the risk of developing diabetes also rises with sustained elevated blood glucose levels [10,12].

Studies suggest a possible association between BED and higher HbA1c, even in individuals without diagnosed diabetes [13]. The reasons behind this connection are still being explored, but several factors might be at play. Binge episodes characteristic of BED often involve significant calorie intake, potentially leading to blood sugar spikes. Additionally, the irregular eating patterns associated with BED can disrupt the body’s natural blood sugar regulation mechanisms. Furthermore, the emotional distress often present in BED might contribute to elevated blood sugar levels through stress hormones [14]. It is important to note that not everyone with BED will have high HbA1c, and other factors like genetics and overall diet also play a role. However, this potential link underscores a broader connection between BED and metabolic health. HbA1c, being a marker of chronic hyperglycemia, might be a potential indicator for the increased risk of developing BED. While the exact cause-and-effect relationships remain under investigation, these findings suggest that HbA1c might not only reflect elevated blood sugar due to BED but also serve as a potential marker for the presence of BED itself, given its association with the broader MetS risk profile. Although other studies have investigated the link between HbA1c and the MetS, most of these investigations have been carried out in industrialized nations, and very few of these investigations have examined the subjects’ glucose tolerance levels [6]. These studies indicate that HbA1c may be able to predict individuals at risk of developing MetS at levels below those associated with diabetes in normoglycemic first-degree relatives of patients with type 2 diabetes. [6]. Hyperglycemia levels appear to be a significant area for future study, as insulin resistance has been proposed as a unifying underlying mechanism of MetS [15]. Hb1Ac should, however, be considered when calculating MetS risk, according to recent research [16]. Hb1Ac may therefore indicate a crucial biological mechanism that contributes to MetS [17,18,19,20].

## 5. Increased BMI in BED Patients Contributes to MetS

Variations in BMI account for a considerable portion of the effect of binge eating on increasing the risk of MetS. Available research indicates that binge eating causes weight gain over time [21]. This has been proposed because BED has been found to be strongly associated with an increased probability of acquiring MetS [2,5]. However, other findings suggest that there may be additional contributors to cardiovascular risk [9]. BED, at all BMI levels, has been found to be associated with a higher risk of hypertriglyceridemia. Other research indicates that BED is linked with hypertension [10]. However, another study found that the association between BED and hypertension was shown to be statistically insignificant [22]. Both hypertriglyceridemia and hypertension are contributors to cardiovascular disease [23]. A biological association was found between hypertriglyceridemia and BED in a population of children and adults [10,22].

There is proof that food ingested during binge eating episodes is often of a lipidic nature and even more often of a carbohydrate nature [24]. Research has previously demonstrated carbohydrate-induced hypertriglyceridemia [9]. A dose–response relationship has been demonstrated through research. The consumption of higher free sugar intake was associated with increased levels of total triglycerides. Furthermore, consumption of refined grain starch was found to increase the risk for cardiovascular diseases (CVDs). The study revealed that the consumption of non-free sugars and whole grain starch was found to be protective for CVD. The consumption of nutrient-dense carbohydrates is strongly associated with an increase in plasma triglycerides [25]. According to studies, a diet with a carbohydrate base is linked to dyslipidemia and high blood pressure in both children and adults [26]. Triglycerides have also been proposed as a potential cause of leptin resistance [15]. This may imply that metabolic abnormalities brought on by BED potentially accelerate weight gain, increasing the chance of developing MetS over time [16].

## 6. Oxidative Stress Contributes to MetS in BED Patients

Inflammatory changes are believed to be a key mechanism for the emergence of metabolic disorders, and huge quantity of food consumed quickly can also increase oxidative stress as well as inflammatory stress. An increase in the production of reactive oxygen species (ROS) is associated with the consumption of glucose. The rise in ROS levels is due to multiple dynamics. Mononuclear cells along with inflammation contribute to the rise in ROS levels by upregulation of the nuclear factor (NF-κβ). Additionally, consumption of glucose contributes to an increase in activator protein-1 (AP-1) which further leads to an increase in ROS levels. Early growth response (Egr-1) binding is also noted following consumption of glucose. Additionally, glucose intake provokes an increase in the levels of tissue factor as well as an overexpression of matrix metalloproteinase-2 (MMP), which further contributes to a rise in ROS levels. Hence, glucose consumption increases inflammatory stress [27]. MMP-2, MMP-9, and tissue. factor plasma concentrations all rise together. A moderately substantial, high-fat, high-carb dinner (900 kcal) can stimulate oxidative stress as well as inflammation in healthy adults in a more recent study [27]. When glucose-intolerant patients and diabetic patients are exposed to a meal challenge test to assess how the body processes glucose following food intake, hyperglycemia can be noted. It is obvious that eating food high in glucose, fat, and macronutrients causes oxidative stress, which is pro-inflammatory and prothrombotic [27].

A study demonstrated that a big (1800 kcal) high-fat, high-carbohydrate dinner in obese individuals provoked an increase in mononuclear cell ROS generation. Additionally, a rise in p47phox expression was recorded. Intranuclear NF-κβ binding also increased. A rise in the plasma MMP-9 concentrations was also noted. Additionally, obese patients showed an increased degree of oxidative stress and inflammation at baseline (albeit not statistically significant for all indices) over the course of the three-hour period. Furthermore, after the meal, plasma levels of MMP-9 and the NADPH oxidase subunit p47phox were considerably greater in obese patients compared to lean subjects. This shows that obese people have a lower ability than lean individuals to tolerate oxidative and inflammatory stress challenges [27]. Overweight people experience increased oxidative and inflammatory stress when they are fasting, and this stress will only increase with additional dietary challenges [27].

Evidence suggests that obesity is closely linked to a persistent inflammatory state. It is characterized by elevated levels of circulating inflammatory factors. For instance, increased levels of high-sensitive C-reactive protein (hs-CRP) and tumor necrosis factor-a (TNF-α) have been recorded. Additionally, a rise in the levels of interleukin-6 (IL-6) is also noted [28,29]. Conversely, weight reduction is associated with a decrease or normalization of these markers. These inflammatory markers are known to causally contribute to obesity-related conditions. For instance, a higher occurrence of insulin resistance, type 2 diabetes, cardiovascular risk is observed with the elevation of the previously mentioned inflammatory markers [28]. Observations reveal that obese individuals with BED also exhibited heightened levels of inflammatory markers such as: hs-CRP, erythrocyte sedimentation rate (ESR), and white blood cell (WBC) counts. Cardiovascular disease and T2DM have a strong association with obesity due to the chronic state of subclinical inflammation [30,31,32,33,34]. An elevation in hs-CRP, in correlation with insulin resistance, is a predictor for cardiovascular risk [35]. Obesity-induced inflammation arises from multiple factors. For example, the buildup of lipids in adipose tissue and the expansion of fat mass contribute to the worsening of the inflammatory state. This, in turn, triggers the production of proinflammatory cytokines and chemokines, leading to inflammation within adipose tissue. Additionally, obesity, atherosclerosis, T2DM, and insulin resistance have been linked to increased levels of uric acid [36]. This association is potentially due to pro-oxidant and pro-inflammatory effects interfering with glucose uptake. Increased levels of uric acid have been noted in cardiovascular diseases patients as well as those with subclinical organ damage [37]. The rapid consumption of large quantities of food leads to an increase in both oxidative stress and inflammatory stress [27,38]. Therefore, metabolic disturbances may arise as a result of inflammatory changes acting as an underlying causal mechanism.

A study was conducted to assess the reaction of inflammatory markers to binge eating in different areas of the brain. IL-18/IL-18 receptor were downregulated in the hypothalamic preoptic. It was additionally recorded in the anterior–tuberal sections [39]. Furthermore, an upregulation of the inhibitor of the pro-inflammatory cytokine IL-18, IL-18BP, was noted. Additionally, the binding chain of the IL-18 receptor was recorded to have a decrease in its expression. Moreover, the hypothalamic anterior–tuberal region showed a three-fold increase in iNOS expression [39]. When the conditions were changed and food was restricted during the stressful estrus phase, the expression of IL-18 increased, however that of iNOS attributed no significant changes [39]. Study limitations include a small sample size and data derived from a cross-sectional study of obese individuals. However, the study included all patients who consecutively sought weight loss treatment. Another limitation is that insulin sensitivity was assessed using the HOMA-IR index. Although the euglycemic–hyperinsulinemic clamp method is considered the gold standard for measuring insulin sensitivity, it is impractical for large studies due to its time-consuming and costly nature [39]. Food intake was suppressed by the direct action of cytokines (IL-1, IL-6, IL-18, TNF-α, and IFN-γ) in the central nervous system [40] through the impact of hypothalamic neurons which regulate eating behavior. IL-1, IFN-γ, and TNF-α influence peripheral signals to the feeding centers by influencing the firing rate of neurons in the lateral hypothalamus that are sensitive to glucose [41]. Indirect actions of cytokines were discussed by Corcos and colleagues [42]. More specifically, IL-1β was shown to have a beneficial effect on plasma catecholamine levels, which in turn lead to decreased food intake [43].

There has been a rising interest in the association between pro-inflammatory cytokines and BED. They are seen to respond intensely to glucocorticoids and stressful stimuli and, thus, regulate feeding behavior [44,45]. Moreover, feeding schedules and stress levels are subject to inflammatory mediators [46,47]. When assessing cases of BED, significant alterations are observed in the levels of circulating cytokines and their secretion from peripheral blood mononuclear cells (PBMCs) [8,42,48]. Moreover, binge eating animals demonstrated alterations in the anterior–tuberal hypothalamus, exhibiting a downregulation of the IL-18/IL-18R system and an induction of iNOS expression. Specifically, in adults, food intake, metabolism, and adiposity were regulated by the anorexigenic cytokine IL-18. A reduced effectiveness of IL-18, either through knockout of the cytokine or its receptor [49,50], or by overexpression of the IL-18BP antagonist [51], led to several abnormalities and the development of MetS in genetically modified mice. Hyperphagia precedes weight gain in both genetically modified male and female mice with IL-18 deficiency. Moreover, both knockout and wild-type mice exhibited a decrease in food intake following central administration of IL-18 [51]. Clinical studies on eating disorders have highlighted an impairment in the IL-18/IL-18R system. Conversely, circulating levels of IL-18 in the periphery were found to be increased, correlating with BMI in obese women [52] and overweight adolescents [53]. In response to IL-18, obese patients demonstrated a decreased production of IFN-γ from PBMCs.

A study analyzed plasma levels of C-reactive protein (CRP), TNF-α, IL-6, leptin, ghrelin, and glucagon-like peptide-1 (GLP-1) in individuals with BED compared to those without BED. The results revealed that individuals with BED exhibited significantly higher rates of irregular eating habits, increased depressive symptoms, and elevated levels of leptin, CRP, and TNF-α [54]. This study has a few limitations that should be considered when interpreting the findings. One key limitation is the nature and size of the sample, which may limit the extent to which these results can be generalized. Additionally, the decision to exclude patients who are using psychotropic medications could be seen as a limitation, as it may have left out individuals with severe psychiatric conditions. However, many psychotropic drugs are known to cause metabolic changes, including weight gain and hyperglycemia. As a result, these medications could act as confounding factors when examining the connection between BED and metabolic hormone levels. Since the study uses a cross-sectional design, it cannot establish causal relationships. Another limitation is that hormone measurements were only taken once in the morning, which does not account for the well-known circadian rhythms that influence hormone secretion [54].

Research on animals suggests that inflammation can disrupt the regulation of appetite and satiety by impairing gut–brain axis communication, leading to reduced sensitivity to ghrelin and GLP-1 [55,56]. Chronic low-grade inflammation is a hallmark of obesity. The central nervous system produces inflammatory cytokines, which directly affect hypothalamic neurons that regulate appetite and eating behaviors [39]. Binge eating may contribute to inflammation, potentially linked to the substantial amounts of food consumed during episodes [57]. Overnutrition induces a pro-inflammatory phenotype characterized by increased TNF-α release from adipocytes [58,59]. Long-term low-grade inflammation associated with increased weight and obesity can lead to reduced leptin sensitivity, impairing appetite regulation [60]. BED patients exhibited signs of low-grade systemic inflammation, evidenced by higher levels of CRP and TNF-α compared to the non-BED group [29]. These findings align with previous research on BED patients with obesity. The BED group also demonstrated elevated levels of GLP-1, a satiety hormone, in contrast to the non-BED group. Inflammatory stimuli have been shown to increase GLP-1 secretion [61]. Elevated levels of leptin and CRP in the BED group suggest that pro-inflammatory cytokines and heightened inflammation may influence leptin, which, in turn, drives GLP-1 synthesis [62]. This interplay may contribute to the higher GLP-1 levels observed in BED patients, reflecting the complex interactions between inflammatory markers and hormonal regulation in this population.

## 7. Genetic Factors Linked to MetS in BED Patients

Genetic factors that are unrelated to obesity are seen to influence BED [3,4]. Thus, it is possible that such factors could raise MetS risk in independent ways, unrelated to high BMI and/or frequent BE behavior [3,4]. For instance, in addition to empirical data showing that mild to extreme cases of BED were statistically significant in mood disorders [2], a connection to MetS and shared characteristics with BED have also been established [2]. These findings are consistent with earlier research that has demonstrated a significant genetic additive effect in both obesity and binge eating [5], as well as a small genetic overlap between the two phenotypes [10]. In addition, binge eating is a behavior that is only modestly influenced by shared surroundings [10,27,63].

When compared to the increasingly common proopiomelanocortin (POMC) and leptin receptor (LEPR) gene mutations, melanocortin-4 receptor (MC4R) gene polymorphisms are seen to be linked to BED and obesity [6]. A retrospective analysis was done to compare eating habits, esophagogastric pathology, the prevalence of the MetS, post-surgery weight loss, as well as comorbidities among gene variations carriers and non-carriers with and without BED [6]. MC4R variants were linked to BED and less MetS improvement, while POMC and LEPR variants showed no such associations. [22]. Overall, the results suggest that individuals with MC4R gene variations exhibit BED, characterized as severe and associated with MetS, compared to matched individuals with severe obesity who are non-carriers of gene abnormalities and do not engage in binge eating [64]. Subjects with the MC4R mutation, which is a potential gene associated with the regulation of eating behavior, exhibit binge eating as one of their primary behavioral traits [15].

Hunger has been associated with multiple genes. The nutritional status conveying adipocyte-derived hormone leptin binds to the leptin receptor [16]. This triggers POMC neurons to release melanocyte-stimulating hormone. The anorectic effects of the hormone melanocyte-stimulating hormone (MSH) are influenced by its attachment to its receptor, MC4R [17]. The MC4R gene has been associated with the development of obesity in 4% of individuals with a BMI over 35 [18]. In contrast, rare case reports highlight monogenic obesity linked to leptin, LEPR, and POMC genes. To date, approximately 30 distinct MC4R mutations have been associated with obesity [19]. BED independently affects 2% to 5% of non-obese individuals; however, in 30% to 90% of obese patients, this suggests that alterations in the leptin-melanocortin system may contribute to binge eating in individuals with severe obesity [20].

Subjects with and without leptin-binding domain mutations in LEPR exhibited similar BED occurrence rates, despite having serum leptin levels within the normal range [64]. Furthermore, no mutations were identified in the POMC-coding region for melanocyte-stimulating hormone, suggesting that the most likely cause of BED in individuals with MC4R mutations is MC4R dysfunction [9]. Greater food craving measures are linked to the C allele of the rs17782313 MC4R marker, a variable that might be the base of the association between BMI and the genetic variation. These encouraging findings were hypothesized based on research conducted on adults of European ancestry [26]. The Src-homology 2B adaptor protein 1 (SH2B1) gene on chromosome 16p11.2 encodes a scaffold protein with four isoforms (α, β, δ, γ) differing only in their COOH termini. It interacts with various receptor tyrosine kinases, cytokine receptors, and Janus kinase (Jaks) complexes involved in signaling pathways for leptin, insulin, IGF-1, and other growth factors. These pathways regulate food intake and energy expenditure, linking SH2B1 to obesity development [65,66].

The SH2B1 gene, recently identified as a candidate for non-syndromic monogenic obesity, encodes an intracellular adaptor protein involved in signaling pathways downstream of receptor tyrosine kinases, including leptin, BDNF, and insulin receptors [22]. The critical roles of these pathways in regulating food intake, energy expenditure, and glucose homeostasis underscore the importance of SH2B1 in obesity development and insulin resistance [23]. Loss-of-function SH2B1 mutations have been strongly linked to obesity based on data from genomic structural variation studies, GWAS, and animal models. Rare deleterious mutations in SH2B1 can lead to non-syndromic monogenic obesity, characterized by hyperphagia and severe early-onset obesity [2].

## 8. Neuropathology of BED

**Figure 1:**
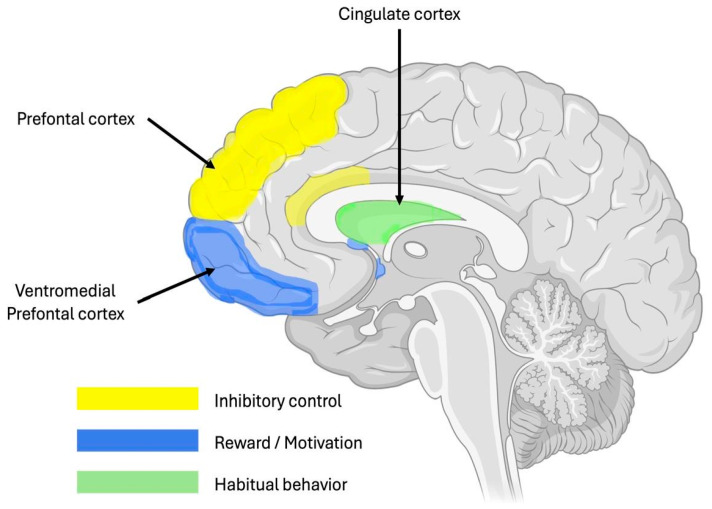
Figure illustrating the implication of brain circuitry in BED. Created in https://BioRender.com (accessed on 10 January 2025).

The neurological underpinnings of BED are gradually becoming clearer. Individuals with BED exhibit increased impulsivity, compulsivity, and altered reward sensitivity, as well as biases toward food and impaired cognitive function [2,9,26]. As illustrated in Figure 1, brain areas such as the ventral striatum, which governs goal-seeking behaviors, motivation, and reward sensitivity, are implicated in the neurobehavioral profile seen in BED. The dorsal striatum, which supports compulsive and habitual behaviors, and the prefrontal cortex, responsible for executive function, are also involved [23,26]. Moreover, the insula, which plays a key role in bodily awareness, cognitive control, taste sensation, and appetite regulation, may contribute to BED, much like its involvement in addiction and impulsivity disorders [15,26]. Additionally, multiple neurotransmitter systems are involved in the neuropathological basis of BED, including the dopaminergic, serotonergic, cholinergic, noradrenergic, GABAergic, opioidergic, and glutamatergic pathways [9,15]. However, neuroimaging studies and animal research suggest that the onset of BED is likely due to alterations in dopamine function [17,26].

**Figure 2:**
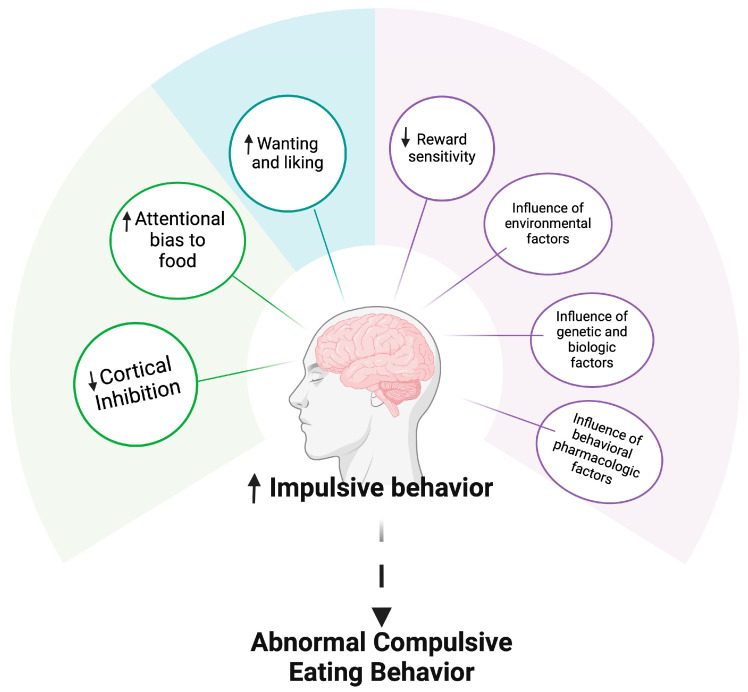
Scheme illustrating the implication of brain circuitry in BED. Upward arrows indicate an increase, while downward arrows indicate a decrease. Created in https://BioRender.com (accessed on 12 February 2025).

One theory suggests that binge eating is caused by an imbalance between the indirect striatopallidal pathway and its associated D2-like receptors, which are responsible for behavioral flexibility, and the direct striatonigral output pathway and its associated D1-like receptors, which are responsible for reward [2,17]. Animal models of binge eating show lower levels of D2-like receptors, indicating probable hypofunction of the indirect pathway compared to the direct pathway, which may underlie the uncontrollable eating behaviors seen in BED, as illustrated in Figure 2 [17]. The transition to binge eating behaviors is thought to involve a shift from impulsive, dorsal–striatal reward-based food consumption to a ventral–striatal reward-based mode [2]. This change is likely due to reduced dopamine release in the striatum, decreased reward sensitivity, and impaired cortical inhibition of reward-related food intake [26]. Dysfunction in the indirect and direct striatonigral pathways is believed to contribute to compulsive eating [26]. The preservation of direct pathway function linked to dopamine D1-like receptors promotes binge eating behaviors, while diminished dopamine D2-like receptor expression is associated with low indirect pathway function [17]. In animal models, chronic dopamine release can lead to this imbalance, with restricted access to appetizing food altering dopaminergic neurotransmission and contributing to binge eating [17]. The Taq1A polymorphism on the ANKK1 gene, which affects the brain’s dopamine system, is frequently studied as a marker for these behaviors [17]. The mesocorticolimbic dopamine system plays a central role in controlling behaviors triggered by natural reinforcers like food or substances like cocaine, with a strong association between addiction and dopamine receptor genes, particularly DRD2 and ANKK1 [17].

The opioid system plays a key role in promoting eating behaviors by enhancing the hedonic qualities of food and regulating reward. The A1 allele of the Taq1A polymorphism is linked to a reduction in dopamine D2 receptors, affecting reward signaling and is considered a ‘loss-of-function’ allele [17]. In contrast, the G allele of the A118G polymorphism in the OPRM1 gene is linked to increased reward sensitivity, making it a ‘gain-of-function’ allele [17]. Studies show that specific gene combinations (A1+ and G- vs. A1- and G+) are associated with BED in obese individuals [3,4]. Recent research also indicates that the Taq1A and C957T genotypes, which enhance dopamine neurotransmission, are strongly associated with BED [2]. These findings suggest that BED may be a subtype of obesity marked by heightened sensitivity to rewards.

The A2/A2 genotype was associated with increased BED sub-phenotypes, including binge, hedonic, and emotional eating, as well as food cravings, while the T/T genotype was linked to higher frequency and severity of binge eating [2]. It has been proposed that combining related genetic variants into a composite polygenic index may account for the variance in phenotype and provide statistical significance, especially in studies with smaller sample sizes [16]. Using this approach, six functional markers across four dopamine genes (DRD2, ANKK1, COMT, DAT1) revealed that the BED group had a higher multi-locus genetic profile score than obese individuals without BED, suggesting that those with BED have stronger dopamine signaling in the brain’s striatum and are more responsive to rewards [16]. These findings align with previous research indicating that BED may be a subtype of obesity characterized by heightened reward sensitivity [2,16]. It is further proposed that dysfunction in the dorsal striatum, coupled with reduced prefrontal cortical control, may lead to compulsive, habitual behaviors, including binge eating [16].

## 9. BED and Food Insecurity

Numerous studies have established a strong connection between food insecurity (FI) and various adverse effects encompassing physical, emotional, and behavioral aspects [67]. FI is described as the limited ability to access sufficient and nutritious food necessary for a healthy lifestyle, primarily due to financial constraints or lack of other essential resources [68]. While FI has been shown to influence eating habits, the specific impact on behaviors like binge eating is not well explored. FI remains a significant public health issue, as emphasized by the Healthy People 2030 objectives. While FI rates showed some improvement between 2018 and 2020, recent studies indicate an increase in FI due to the COVID-19 pandemic [69]. The prevalence of eating disorders and related symptoms has also surged during the pandemic [70,71]. Since the global health crisis declaration in March 2020, numerous studies have examined the pandemic’s impact on food availability, access, and utilization [72,73]. Movement restrictions, disruptions in food supply chains, and food price volatility have threatened food and nutrition security worldwide [73]. However, the pandemic’s effects have not been uniform; low-to-middle-income countries and those experiencing ongoing conflicts or humanitarian crises have been especially vulnerable to these challenges [74].

Families experiencing FI often face irregular eating patterns, known as the ‘feast-or-famine’ cycle, where periods of food scarcity followed by abundance lead to restrictive eating followed by overeating. This cycle may contribute to the onset of binge eating behaviors. Additionally, the stress of food scarcity activates the hypothalamic–pituitary–adrenal axis, triggering the release of cortisol, a hormone linked to binge eating [75]. FI has been associated with an increased likelihood of binge eating, as well as the development of BED and its subclinical form, other specified feeding and eating disorder-binge eating disorder (OSFED-BED) [69]. Factors such as fluctuating food availability and stress from food scarcity may contribute to this relationship, particularly during childhood and adolescence. For example, participants in the Supplemental Nutrition Assistance Program often face food scarcity before the month’s end, leading to restrictive eating and later binge eating [76,77]. A recent meta-analysis also found that FI is linked to higher fast food consumption, which may increase the risk of binge eating due to the affordability and caloric density of these foods [78,79,80].

FI, a key contributing factor to mental health disorders such as depression and anxiety, is associated with unfavorable emotional, cognitive, and behavioral effects, potentially increasing susceptibility to the distress and humiliation associated with a diagnosis of BED and OSFED-BED [69,81,82,83,84]. Moreover, negative emotions are widely recognized as triggers for binge eating, as outlined in affect regulation theories. In some instances, binge eating might serve as a coping strategy to reduce emotional distress. Additionally, FI is often linked to guilt and self-criticism, which can further heighten the likelihood of binge eating episodes. [85,86]. Additionally, FI is closely tied to socioeconomic status and poverty, which may influence the development of BED. One study found that general financial difficulties, beyond FI, also played a role in binge eating, highlighting the need to address financial challenges when developing interventions for affected populations [87]. For example, low-income individuals may face additional barriers, such as limited access to food, which can worsen FI.

Individuals experiencing FI often practice cognitive dietary restraint to manage resources for other household members or stretch their food supply over time, which can increase the likelihood of binge eating [88]. The stress associated with FI may also activate cortisol production pathways, a key risk factor for binge eating and the consumption of highly palatable foods [75,89]. Studies show that adults in the FI group are 1.66 times more likely to engage in binge eating and 2.70 times more likely to develop BED compared to food-secure adults [90]. The relationship between FI and binge eating is likely influenced by limited access to food, leading to feelings of dietary restraint, anxiety about eating, restricted food choices, and preoccupation with food [88,91]. These factors can contribute to overeating and loss of control when food becomes available [92]. Individuals facing FI are more likely to live in areas known as ‘food deserts,’ where access to healthy foods such as supermarkets and large grocery stores is limited, and ‘food swamps,’ areas with a high density of stores offering unhealthy, energy-dense foods like fast food restaurants and convenience stores [93,94,95]. These environments contribute to binge eating behaviors, as high-calorie, highly palatable foods are often more accessible and affordable than healthier options, particularly for low-income individuals [96,97,98,99]. Exposure to such environments may trigger food cravings and lead to overeating, potentially contributing to the development of MetS [97,98].

## 10. The Role of Trauma in the Development of BED and MetS

It is essential to consider trauma as a potential shared risk factor in the relationship between BED and MetS. There is no unanimous agreement on the conceptual definition of trauma. Some definitions, including that of the DSM-5-TR, are very narrow in scope, encompassing only serious events such as life-threatening accidents, death, or sexual abuse [1]. However, in research studies, the concept of trauma is frequently broadened to include ’adverse childhood experiences’ (ACEs) (e.g., interpersonal loss, parental maladjustment, maltreatment, and family economic adversity) [100] or ’life adverse experiences’ (LAEs) (e.g., emotional abuse, physical abuse, sexual abuse, sexual harassment, rape, peer bullying, witnessing domestic violence, and serious accidents) [101]. A growing body of research suggests that trauma is linked to both BED and MetS separately, raising the possibility that it may contribute to shared causal pathways or even shed light on underlying pathophysiological mechanisms connecting these conditions.

Trauma has long been implicated in the development of general psychopathology, with early adversity being a well-established risk factor for a range of psychiatric disorders, including depression, anxiety, and post-traumatic stress disorder (PTSD) [100,102]. Eating disorders, including BED, are particularly linked to general forms of trauma, with individuals who have experienced ACEs [103] and LAEs [104,105,106] displaying significantly higher rates of disordered eating behaviors. Research has also explored the effects of specific forms of trauma on the development of eating disorders. For example, veterans who endured military sexual trauma (MST) had a two-fold increased likelihood of developing an eating disorder compared to veterans with no history of MST [107], with this association being especially pronounced among male veterans. The temporal relationship between trauma and the onset of eating disorders has also been explored, with studies reinforcing the precedence of trauma over the development of eating disorders [108]. This suggests that trauma is a risk factor for disordered eating rather than the other way around. While literature clearly demonstrates a strong correlation between eating disorders and trauma, it does not yet provide a definitive explanation for this relationship. Many hypotheses have been proposed but are yet to be systematically evaluated.

Similarly, trauma has been associated with the development of MetS. Chronic stress, adverse life experiences, and childhood trauma have been shown to contribute to the development of MetS by influencing changes in waist circumference, triglyceride levels, high-density lipoprotein (HDL) cholesterol, blood pressure, and glucose levels [109,110]. Notably, the type of adversity, sex, number of traumatic experiences, and sleep quality all serve as relevant mediators affecting the relationship between trauma and MetS. While emotional and physical abuse elevate the risk of developing MetS in both men and women, sexual abuse is a risk factor exclusively for women [111]. Moreover, higher cumulative exposure to abuse, along with poor sleep quality, further increases the likelihood of MetS in both sexes [111]. The literature linking trauma separately to BED and MetS suggests that trauma may serve as a connecting factor between the two conditions. Individuals with a history of adverse childhood experiences not only exhibit higher rates of BED but also show an increased prevalence of MetS-related health concerns. Future research should aim to further elucidate these relationships and their etiological underpinnings, potentially leading to more targeted prevention and treatment strategies that address both metabolic and psychiatric vulnerabilities in individuals with trauma histories.

## 11. The Complex Interplay Between Binge Eating Disorder and Metabolic Syndrome: Etiological Factors and Clinical Implications

BED is significantly linked to MetS, which includes conditions like hypertension, obesity, type 2 diabetes, and dyslipidemia. The connection between MetS and BED is well-established, with behavioral, genetic, biological, neurological, and pharmacological factors all playing a crucial role in the relationship. Table 2 summarizes the factors associated with the prevalence of MetS in individuals with BED. Biological, genetic, neurologic and behavioral factors greatly contribute to the etiology of BED. These factors intertwine and exert additive effects when present together. For instance, the consumption of a high-fat and high-carb diet is a behavioral factor that leads to the development of biological factors such as oxidative stress and inflammatory stress, which in turn contribute to the etiology of BED. Additionally, neurological factors, such as increased brain dopamine signaling, contribute to the development of BED, with this being considered a subtype of obesity that is sensitive to reward. Genetic factors, such as the Taq1A polymorphism on the ANKK1 gene, enhance the effect of the dopaminergic system, further potentiating the link between various factors. Food insecurity has also been found to contribute to the etiology of BED. Families experiencing food insecurity face irregular eating patterns, which can lead to the onset of BED behaviors. Additionally, trauma plays a role in the development of these disorders [2,16,22,23,64].

## 12. Conclusions

BED is a complex condition with profound metabolic and psychological consequences, significantly contributing to the development and progression of MetS. The interplay between behavioral, genetic, biological, and neurological factors underscores the need for a multidimensional approach to diagnosis and management. Beyond its direct metabolic impact, BED is influenced by socioeconomic disparities, food insecurity, and broader systemic factors, necessitating targeted public health strategies. Given the substantial burden of BED and its association with chronic diseases, early identification, personalized treatment interventions, and integrative management strategies are essential. Further research is needed to elucidate the precise mechanisms linking BED and MetS, optimize therapeutic approaches, and improve long-term outcomes for affected individuals. Addressing BED within the framework of metabolic and public health concerns can significantly enhance prevention and treatment efforts, ultimately reducing the risk of chronic disease and improving overall well-being.

## Figures and Tables

**Table 1:** Diagnostic Criteria for BED.

	Duration	Associated Symptoms	DSM-5 Traits
BED Episode	BED episodes must be noted for at least three months. The occurrence must be recorded as a minimum of one per week [2].	Absence of compensatory behaviors such as purging. Distress must also be associated with each BED episode [2].	Must fulfill the DSM-5 criteria. A minimum of three of the following traits is required [5]: Patient eating at a faster pace compared to their baseline speed. Eating until discomfort is reached. The urge to eat despite not feeling hungry. Preference for eating alone due to feelings of shame, often associated with consuming large amounts of food. Development of feelings of guilt, shame, and self-disgust. Such feelings develop following a binge eating episode

**Table 2:** Factors associated with the prevalence of MetS in BED patients and their outcomes.

Behavioral Factors	Biological Factors	Genetic Factors	Neurological Factors
High-fat and -carb diet (results in an increase in oxidative stress as well as engendering inflammatory stress through rise of ROS)	Oxidative stress leading to a rise in mononuclear cell ROS generation, p47^phox^ expression, intranuclear NF-κβ binding, and plasma MMP-9 concentrations	Rise in Hb1Ac levels (serves as a potential marker for BED)	Brain areas such ventral striatum, dorsal striatum, prefrontal cortex, and the insula (dysfunction in pathways within these regions is believed to contribute to compulsive and uncontrollable eating behaviors observed in BED)
Decreased weight cycling episodes		Taq1A polymorphism on ANKK1 gene (results in the enhancement of the effects of the dopaminergic system making BED a possible subtype of obesity marked by heightened sensitivity to reward)	Increased brain dopamine signaling (results in the enhancement of the effects of the dopaminergic system making BED a possible subtype of obesity marked by heightened sensitivity to reward)
Regular meal skipping	Inflammatory stress leading to elevated levels of hs-CRP, IL-6, and TNF-α, and uric acid (results in increased insulin resistance, a low-grade inflammation associated with increased weight and obesity can lead to reduced leptin sensitivity, impairing appetite regulation)	SH2B1 loss of function mutations (results in hyperphagia, insulin resistance, and severe early onset obesity)	
	Accumulation of visceral adipose tissue and downregulation of the IL-18/IL-18R system and an induction of iNOS expression (results in hyperphagia and thus weight gain).	MC4R polymorphisms (results in dysregulation of eating behavior)	

## Data Availability

This study is a review article and does not involve the generation or analysis of new data. All data supporting this work are derived from previously published studies, which are appropriately cited in the manuscript.
